# High lability of sexual system over 250 million years of evolution in morphologically conservative tadpole shrimps

**DOI:** 10.1186/1471-2148-13-30

**Published:** 2013-02-05

**Authors:** Thomas C Mathers, Robert L Hammond, Ronald A Jenner, Thorid Zierold, Bernd Hänfling, Africa Gómez

**Affiliations:** 1 School of Biological, Biomedical and Environmental Sciences, University of Hull, Hull, HU6 7RX, UK; 2 Department of Biology, University of Leicester, University Road, Leicester, LE1 7RH, UK; 3 Department of Life Sciences, The Natural History Museum, Cromwell Road, London, SW7 5BD, United Kingdom; 4 Museum für Naturkunde Chemnitz, Moritzstrasse 20, D-09111, Chemnitz, Germany

**Keywords:** Androdioecy, Character evolution, Sexual system, Notostraca, Phylogeny

## Abstract

**Background:**

Sexual system is a key factor affecting the genetic diversity, population structure, genome structure and the evolutionary potential of species. The sexual system androdioecy – where males and hermaphrodites coexist in populations – is extremely rare, yet is found in three crustacean groups, barnacles, a genus of clam shrimps *Eulimnadia*, and in the order Notostraca, the tadpole shrimps. In the ancient crustacean order Notostraca, high morphological conservatism contrasts with a wide diversity of sexual systems, including androdioecy. An understanding of the evolution of sexual systems in this group has been hampered by poor phylogenetic resolution and confounded by the widespread occurrence of cryptic species. Here we use a multigene supermatrix for 30 taxa to produce a comprehensive phylogenetic reconstruction of Notostraca. Based on this phylogenetic reconstruction we use character mapping techniques to investigate the evolution of sexual systems. We also tested the hypothesis that reproductive assurance has driven the evolution of androdioecy in Notostraca.

**Results:**

Character mapping analysis showed that sexual system is an extremely flexible trait within Notostraca, with repeated shifts between gonochorism and androdioecy, the latter having evolved a minimum of five times. In agreement with the reproductive assurance hypothesis androdioecious notostracans are found at significantly higher latitudes than gonochoric ones indicating that post glacial re-colonisation may have selected for the higher colonisation ability conferred by androdioecy.

**Conclusions:**

In contrast to their conserved morphology, sexual system in Notostraca is highly labile and the rare reproductive mode androdioecy has evolved repeatedly within the order. Furthermore, we conclude that this lability of sexual system has been maintained for at least 250 million years and may have contributed to the long term evolutionary persistence of Notostraca. Our results further our understanding of the evolution of androdioecy and indicate that reproductive assurance is a recurrent theme involved in the evolution of this sexual system.

## Background

Plants and animals have evolved a great diversity of sexual systems that range from the extremes of obligatory self-fertilisation to complete outcrossing. Transitions between these sexual systems have long fascinated biologists due to the impacts they have on key parameters such as inbreeding depression, genetic diversity, population structure, genome structure and the evolutionary potential of species [1-7]. Transitions between sexual systems often present tradeoffs between short and long term selective advantages and can have significant connotations for the long-term viability of species. For example, selection for reproductive assurance and colonisation advantage due to mate limitation during range expansions, or as a result of high population turnover in metapopulations, can drive transitions to self-fertilisation strategies [8-10]. These transitions occur despite the deleterious effects of self-fertilisation, which include inbreeding depression, reduction in effective recombination rates and reduction in effective population size [11].

Transitions to androdioecy (AD) – a sexual system where males and hermaphrodites co-occur in varying frequencies in populations, with different levels of self-fertilisation and outcrossing – are extremely rare in plants and animals [12-16]. In animals, AD has only been described in five groups, rhabditid nematodes, the killifish *Kryptolebias marmoratus* and three crustacean groups; barnacles, a genus of clam shrimps *Eulimnadia*, and in the order Notostraca, the tadpole shrimps [17,18]. AD can evolve either through the invasion of males into hermaphrodite only populations, as in barnacles [18,19], or through the replacement of females with hermaphrodites in gonochoric populations (where males and females are found in approximate equality), as in the plants *Mercurialis annua*[20,21] and *Datisca glomerata*[22]. As models to describe the evolution and maintenance of AD only predict its evolution under stringent conditions, AD has historically been considered an unstable, transitional sexual system between gonochorism and hermaphroditism (or vice versa) [12-14,23,24]. This view is borne out by the scarcity of AD in nature [12,17], although recent research in the branchiopod *Eulimnadia* has revealed an unexpected stability of androdioecy [25].

Notostraca, or tadpole shrimps, is a small order of branchiopod crustaceans characterised by a high level of morphological stasis. Fossils dating back as far as the Triassic are almost indistinguishable from contemporary species leading them to be referred to as ‘living fossils’ [26-30]. In contrast, Notostraca has diverse sexual systems, including gonochorism, self-fertile hermaphroditism and AD, with variation occurring on both an interspecific and intraspecific level [31,32]. Remarkably, AD is found in species from both notostracan genera, *Triops* and *Lepidurus*, suggesting that transitions in reproductive system might have evolved repeatedly in the order. Despite this, the evolutionary history of reproductive systems in Notostraca is unknown due to the lack of a resolved phylogeny [33,34], and the poor knowledge of the diversity of the group, partly due to the widespread presence of cryptic species [32,35-38]. Gonochorism has been hypothesized to be the ancestral state in the group, and the evolution of self-fertile hermaphroditism and AD has been linked to reproductive assurance in the context of range expansions, possibly after glacial retreat [8,31,39,40], although this has never been explicitly tested.

Here we combine newly generated and GenBank sequence data to assess Notostraca taxonomic diversity, identifying considerable cryptic diversity, and employ a multigene phylogenetic approach to create a well-supported, global phylogeny of Notostraca. Information on sexual system was compiled and Maximum Parsimony (MP) and model-based Maximum Likelihood (ML) character mapping approaches were used on the phylogeny to investigate sexual system evolution across the order. We also tested the hypothesis that reproductive assurance has driven the evolution of self-fertilisation across Notostraca [8,39]. Taxa found at higher latitudes are likely to have experienced bouts of colonisation during post glacial range expansions, which would select for AD/hermaphroditism. We therefore compared the latitudes that AD/hermaphroditic and gonochoric taxa are found using a phylogenetic *t*-test. Our analyses reveal high levels of reproductive lability with frequent transitions occurring to and from androdioecy. Furthermore, this flexibility is conserved across Notostraca, and may have been maintained for at least 250 million years. Additionally, AD/hermaphroditic taxa are found at significantly higher latitudes than gonochoric ones suggesting that colonisation advantage through reproductive assurance is likely to be involved in transitions between sexual systems in Notostraca.

## Results

### Delimitation of significant taxonomic units

Notostraca is known to contain cryptic species complexes e.g. [35,37] so we first used a cytochrome oxidase subunit one (COI)–based barcoding approach to identify significant taxonomic units (STUs) for inclusion in our phylogeny prior to the multigene analysis. Including available GenBank data and 12 newly generated sequences for this study, 243 Notostraca COI sequences were aligned. We applied a generalized mixed Yule coalescent (GMYC) model to identify independently evolving clusters in our COI dataset, which correspond to STUs. The GMYC model identified 26 STUs (Additional file 1: Figure S1). Uncorrected mean genetic distances in COI between STUs ranged from 2.3% to 24.3%. Four Notostraca lineages did not have COI data available, but are represented by other genes used in our multigene phylogenetic analysis; *T. gadensis, T. cf. granarius* (Tunisia)*, L. bilobatus* and *L. cryptus*. As the species status of these lineages has been confirmed in regional studies of cryptic diversity in Notostraca [35-37,41] they were included as additional STUs for the multigene phylogenetic analysis. In total we recognise 30 STUs within Notostraca.

### Notostracan phylogeny

**Figure 1:**
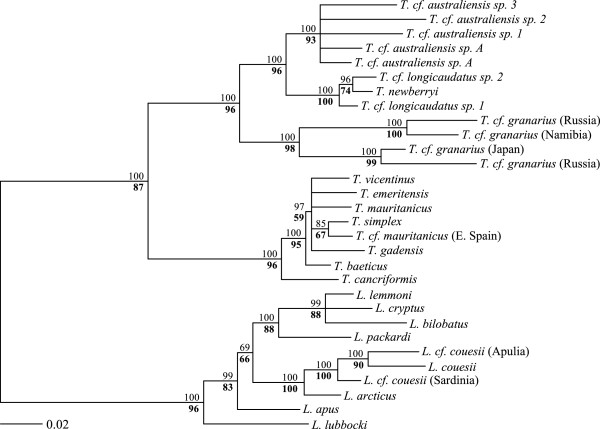
**Phylogenetic relationships in Notostraca based on a multigene supermatrix.** Tree topology shows the best scoring ML tree from the RAxML analysis. Values above nodes show Bayesian posterior probabilities and values below nodes, in bold, show bootstrap support (1000 replicates). Branches with bootstrap support lower than 50 are collapsed. The *Leptestheria* outgroup was removed after rooting.

Phylogeny, based on a concatenated supermatrix of 110 sequences (54 of which were newly generated for this study) from three mitochondrial genes and four nuclear genes for 30 STUs, was inferred by ML and Bayesian Markov-chain Monte Carlo methods. Both methods of phylogenetic reconstruction gave congruent topologies, with most branches having high levels of support (Figure 1). The two recognised notostracan genera, *Triops* and *Lepidurus*, formed highly supported clades. Within *Triops*, four main monophyletic lineages with a strong geographic association (Australian, N American, Palearctic and African/Asian respectively) were highly supported. The analysis supported a close relationship between the *T. australiensis* complex (Australia) and the *T. longicaudatus* complex (North America), and a sister relationship of these with the *T. granarius* complex (Asia/Africa). The *T. cancriformis/mauritanicus* complex (Palearctic) appears as the sister group to the rest of *Triops.* Within *Lepidurus*, *L. lubbocki* (Mediterranean), and *L. apus sensu stricto* (N European) have long branches and are sister species to the rest of *Lepidurus*. Four North American species [35,41]*L. packardi*, *L. cryptus*, *L. bilobatus* and *L. lemmoni*, with narrowly endemic, mostly allopatric distributions in western North America, form a well supported group. *L. arcticus,* a circumpolar species from Arctic and Subarctic regions, forms a sister relationship with a clade containing *L. couesii*, which forms a widely distributed species complex.

### Sexual system assignment

Our literature review identified sexual system data for 22 STUs; 18 of these have at least one barcoded population (either COI, 12S or 16S). In the few cases where an STU does not have a barcode sequence for the same population for which sexual system data is derived (*L. arcticus, L. couesii, L. lemmoni, L. packardi*) these species have been well studied, no further cryptic diversity has been identified and the populations used for sexual system inference fall inside known species ranges (see [35,41,42]). We found two polymorphic STUs; *T. cancriformis* and *T. cf. longicaudatus sp.2*, which include both androdioecious and gonochoric populations, and four androdioecious STUs; *T. newberryi*, *T. cf. australiensis sp. B, L. apus (sensu stricto)* and *L. arcticus* (Table 1 and Additional file 1, Table S6). Sex ratio of populations assigned AD ranged from 0% to 27% males. In addition, either histological data or reproduction in isolation data, or both, confirmed the presence of anatomical hermaphrodites. Fourteen taxa were considered gonochoric on the basis of histology, lack of reproduction in isolation and sex ratio. The quality and quantity of information on sexual system was heterogeneous amongst gonochoric taxa, with actual sex ratios tending to be underreported and histological studies lacking for many taxa. Eight taxa could not be assigned to a sexual system due to an absence of data or equivocal sex ratio.

**Table 1:** Notostraca sexual system information

**STU**	**Sex ratio**	**Reproduction in isolation**	**Ovotestis**	**Sexual system**	**References**
*T. baeticus*	> 45			Gonochoric	[36]
*T. cancriformis*	0 – 53*	Yes**	Yes**	Gonochoric/AD	[26,31,40,43,44]
*T. cf. australiensis sp. 1*				No data	
*T. cf. australiensis sp. 2*				No data	
*T. cf. australiensis sp. 3*				No data	
*T. cf. australiensis sp. A*	> 45		No	Gonochoric	[45]
*T. cf. australiensis sp. B*	< 30		Yes	AD	[45]
*T. emeritensis*	>45			Gonochoric	[36]
*T. gadensis*	36			Equivocal	[36]
*T. cf. granarius* (Japan)	> 45	No		Gonochoric	[26,44,46,47]
*T. cf. granarius* (Namibia)	Even***	No		Gonochoric	[26,37,44]
*T. cf. granarius* (Tunisia)	Even***	No		Gonochoric	[26,37,44]
*T. cf. granarius* (Russia)				No data	
*T. cf. longicaudatus sp. 1*	> 45			Gonochoric	[32]
*T. cf. longicaudatus sp.2*	0 – 68*		Yes**	Gonochoric/AD	[32,48,49]
*T. cf. mauritanicus* (E Spain)				No data	
*T. mauritanicus*				No data	
*T. newberryi*	< 30	Yes		AD	[32,48,50]
*T. simplex*	> 45			Gonochoric	[38]
*T. vicentinus*	> 45			Gonochoric	[36,51]
*L. apus*	< 45		Yes	AD	[26,40,44,52-55]
*L. arcticus*	<45		Yes	AD	[26,40,44,54,56-58]
*L. bilobatus*	35			Equivocal	
*L. cf. couesii* (Apulia)				No data	
*L. cf. couesii* (Sardinia)	>45			Gonochoric	[59]
*L. couesii* (Canada)	>45			Gonochoric	[60]
*L. cryptus*				No data	
*L. lemmoni*	>45			Gonochoric	[56,61]
*L. lubbocki*	>45		No	Gonochoric	[62,63]
*L. packardi*	>45			Gonochoric	[64]

Sex ratio (percent male), ability of ovisac bearing individuals to reproduce in isolation and the presence or absence of ovotestis in ovisac bearing individuals is shown. See Table S5 for detailed information.

* Depending on population. ** Only in populations where sex ratio is < 30%. *** Reported gonochoric with even sex ratio, exact numbers not given.

### Sexual system evolution

**Figure 2:**
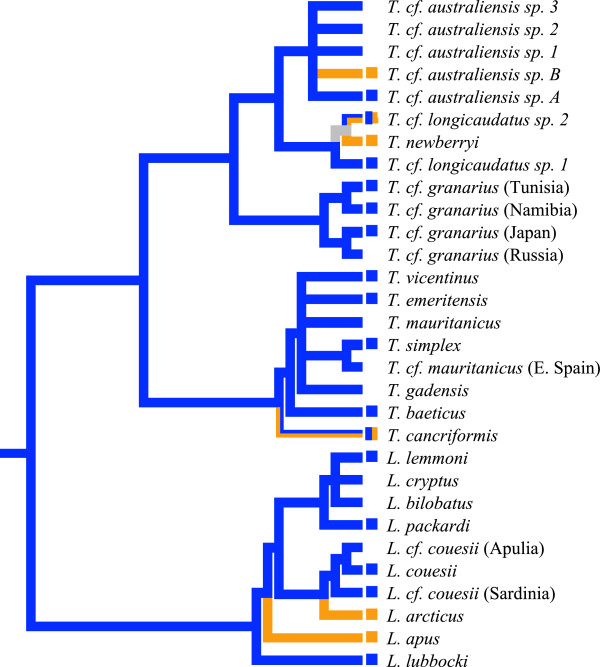
**Maximum parsimony ancestral character state reconstruction of sexual systems in Notostraca.** Sexual system is mapped onto the best scoring ML tree, and indicated by the colour of the square that precedes the taxon names. Blue = gonochoric, Orange = Androdioecy. STUs without squares preceding the taxon name do not have sexual system information or sexual system is equivocal. Bi-coloured squares indicate that both sexual systems are found. Branches are coloured according to MP reconstruction of ancestral sexual systems based on an unordered model with grey branches denoting an equivocal ancestral state assignment. The *Leptestheria* outgroup was removed after rooting and left uncoded for sexual system.

MP reconstruction of ancestral character states infers that gonochorism is the ancestral state of Notostraca (Figure 2). Furthermore, AD appears to have multiple origins in Notostraca having evolved three times in *Triops* and twice in *Lepidurus*. Sexual system is highly flexible across Notostraca and varies even between closely related species (*T. cf. australiensis A* vs. *T. cf. australiensis B*; *T. newberryi* vs. *T. cf. longicaudatus sp.1*) or shows intraspecific variation (*T. cancriformis; T. cf. longicaudatus sp.2*). Model based ML methods showed that a two-parameter model, which allows distinct transition rates for AD to gonochorism and from gonochorism to AD, was a significantly better fit for the data than a model where both transitions have an equal rate (Table 2), or models where transitions were restricted to one direction, either from gonochorism to AD or AD to gonochorism, indicating that in Notostraca changes in sexual system could be bidirectional. Overall, the ML model suggests that transition rates between sexual systems were high and in particular, transitions from AD to gonochorism were more than three times higher than transitions from gonochorism to AD. This result is in striking contrast to the MP results which suggested repeated evolution in the opposite direction, to AD from gonochorism. This indicates that, once evolved, AD may be unstable and likely to revert back to gonochorism. The high rates of change across the order meant that, unlike for the MP analysis where a minimum number of transitions is inferred, ancestral sexual systems for all nodes were equally likely to be either gonochoric or AD. Virtually identical results were achieved using an ultrametric phylogeny constructed in BEAST v1.7.4 [65] with a lognormal relaxed molecular clock both from the full dataset and with a reduced dataset containing only the mitochondrial genes COI, 12S and 16S (see Additional file 1).

**Table 2:** Comparison of models of sexual system evolution in Notostraca

**Model**	**lnL**	***q***_***GA***_	***q***_***AG***_	***p***
Unrestricted	−7.229	23.729	110.640	-
Equal rates	−10.262	5.730	5.730	0.0138
AD to gonochorism only	−16.855	0.000	6.673	< 0.0001
Gonochorism to AD only	−12.0864	1.755	0.000	0.0018

Models of transitions in sexual system across the best scoring ML estimation of Notostraca phylogeny compared using the ML implementation of BayesMultistate. *lnL* = log-likelihood of model, *q*_*GA*_ = transition rate from gonochorism to androdioecy, *q*_*AG*_ = transition rate from androdioecy to gonochorism, *p* = *p*-value for *D* with 1 degree of freedom comparing the restricted models to the unrestricted model.

### Testing the reproductive assurance hypothesis

The mean latitude of STUs where AD is found was significantly higher than for gonochoric ones (phylogenetic *t*-test, *t* = 2.922, *p* = 0.010, d.f. = 17) with an average latitude of 49.329 compared to 37.256. ML value for λ was estimated to be 0.508 and its inclusion significantly improved the likelihood of the model (*D*, *p* = 0.028, d.f. = 1) indicating that the latitude of STUs has moderate phylogenetic signal.

## Discussion

### Sexual system evolution

Our analyses reveal that sexual system is a highly labile trait within Notostraca. The rare sexual system AD has evolved multiple times in both notostracan genera, with MP indicating at least five independent origins across the whole group (Figure 2). This lability is also supported by the model based ML analysis which infers high transition rates between sexual systems. Unlike the MP analysis, the ML analysis did not resolve the ancestral sexual system for any nodes. This suggests that MP may give an oversimplified reconstruction of the evolutionary history of sexual system in Notostraca and highlights the importance of incorporating branch lengths in ancestral state reconstructions [66]. The ML analysis also contrasts with MP by inferring that transitions between gonochorism and AD can occur in both directions as is the case for pedunculate barnacles [18], rather than in a unidirectional manner as implied by MP. As there are no known biological constraints to transitions between sexual systems in either direction there is no reason to discredit the model of sexual system evolution inferred by either method. For example, a transition from AD to gonochorism could occur as a result of selection for outcrossing over selfing, followed by the loss of testis lobes in hermaphrodites. In conclusion, although it is not possible to infer the history of sexual system change in Notostraca with current data or methods of ancestral state reconstruction, our results do display a consistent pattern of high lability of sexual system across Notostraca.

Although flexibility in sexual system has previously been shown in *T. cancriformis*, where a recent transition from gonochorism to androdioecy has occurred [31,43], our results demonstrate that reproductive flexibility is a general feature of Notostraca as a whole. Given the age of the order – well-preserved notostracan fossils date back to the Carboniferous [67] and *Triops* and *Lepidurus* are known from the Permian and Triassic [28,29,68], dating the split in the two genera to at least ~250 million years ago – it can be inferred that reproductive lability has been maintained for at least 250 million years within tadpole shrimps and may have aided their long term evolutionary persistence. Such lability of sexual system in Notostraca contrasts strongly with the dynamics of sexual system evolution in the clam shrimps of the genus *Eulimnadia*, the other branchiopod crustacean where AD is found. In *Eulimnadia*, AD evolved once and has persisted for at least 24 million years, passing through many speciation events [25,69-71]. The contrast in sexual system evolution between both taxa is striking considering that tadpole shrimps and clam shrimps inhabit similar ecological niches [26,72], in some cases occupying the same pool [73,74], and have similar life histories, producing long-lived dormant cysts that survive during adverse periods and also provide the dispersal stage [75-77]. They also have a similar genetic mechanism of sex determination where males are recessive to hermaphrodites in AD populations [78-80]. Finally, in both groups, hermaphrodites are morphologically derived from obligatory outcrossing females, and can either self-fertilise or outcross with males but, unlike in plants or barnacles, are unable to fertilise each other [26,70]. Within Branchiopoda therefore, superficially similar ecological backgrounds and genetic mechanisms of sex determination have resulted in two very different, but equally unusual, evolutionary outcomes for sexual system; stable and conserved in *Eulimnadia* and labile and dynamic in Notostraca. In contrast, the flexibility of sexual system in notostracans resembles that of barnacles, where AD has evolved on several occasions in response to mate limitation, although in this system AD has evolved from a hermaphroditic ancestor rather than a gonochoric one [18,81]. Modelling has explained the persistence of AD in *Eulimnadia* and highlights the importance of the presence of males for limiting inbreeding depression and that a proportion of progeny produced by selfing – those that are homogametic – have reduced fitness due to the expression of sex-linked genetic load [82,83]. In Notostraca, however, these key parameters, along with other factors that contribute to the evolution and maintenance of AD, such as male-hermaphrodite encounter rates and fecundity, have not been investigated. Further research is therefore required to ascertain whether the dynamics of sexual system evolution in Notostraca necessitate the re-evaluation of current models for the evolution of AD in animals. A lack of phylogenetic signal regarding sexual systems in Notostraca suggests that switches in sexual system occur in response to ecological factors. Notostraca share similarities in life history with many plants [8], particularly those that exist in highly dynamic metapopulations, where colonisation of new habitats is a key feature of survival causing selection for reproductive assurance [84-86]. In many cases selection for reproductive assurance has caused a breakdown in plant self-incompatibility systems and the evolution of self-fertile hermaphrodites, which make optimal pioneer genotypes [87]. If reproductive assurance drives the evolution of self-fertile hermaphroditism and hence AD in Notostraca, as has been hypothesised [39,40], we would expect AD STUs to occur in areas which have recently become available for colonisation. These predictions appear to be met in *T. cancriformis,* where hermaphroditic and androdioecious populations occur in previously glaciated areas whereas known gonochoric populations occur in what were previously unglaciated refugia [31]. Furthermore, our results support the reproductive assurance hypothesis for the whole of Notostraca as our phylogenetically informed analysis reveals that AD notostracan STUs occur at significantly higher latitudes than gonochoric ones. These tests indicate that AD STUs occur in areas where habitat disruption through glacial cycles is more likely, providing further support for the role of colonisation advantage and reproductive assurance in a metapopulation as drivers for the evolution of AD.

Highly fluctuating population densities, which result in mate limitation, could also provide an advantage to lineages containing hermaphrodites through reproductive assurance [13,86]. In the case of Branchiopoda, the role of low population densities and mate limitation in the evolution of sexual systems is still poorly understood [82]. Ephemeral ponds are often very small [88] with strong inter-annual fluctuations in branchiopod population density [25], which could provide a context in which self-fertilising hermaphrodites would enjoy an increased fitness relative to females, driving the evolution of AD. Further research on Notostraca species distribution, genetic diversity, metapopulation dynamics and phylogeography will help to understand the underlying factors behind changes in sexual systems in this group.

### Taxonomic implications of notostracan phylogeny

Our results resolve the phylogenetic relationships of Notostraca, supporting the previously disputed [33,34] monophyly of *Triops*. Although the four main lineages in *Triops* coincide with the four species recognised by Longhurst [26] in the last revision of Notostraca, our analyses support previous work revealing that each of these lineages is made up of cryptic species. Each main lineage has maintained a remarkably stable, mostly allopatric geographic distribution (only Japan has representatives of the *T. cancriformis*, *T. cf. granarius* and *T. cf. longicaudatus* complexes, and N Africa shares both *T. mauritanicus* and *T. cf. granarius*). In *Lepidurus*, *L. lubbocki* was found to be the sister species to the rest of the genus and as suggested by Mantovani et al. [89] full species status is warranted. Further cryptic diversity was also identified in the *L. couesii* complex and given that the type locality for *L. couesii* is in N America [41] and the level of divergence with Apulian (S. Italy) and Sardinian lineages, we propose these latter lineages are new species. Overall, although highlighting the need for further research into Notostraca taxonomic diversity, our phylogeny represents an excellent framework for the study of evolutionary processes within the order.

## Conclusions

Our analyses show that sexual systems are extremely flexible in Notostraca, with repeated switches between gonochorism and androdioecy possibly driven by postglacial range expansions. This unconstrained, labile pattern, strongly contrasts with the single origin of androdioecy in the genus *Eulimnadia* despite the similarity of their habitats and life histories and resembles the pattern found in barnacles. Despite the idiosyncratic evolution in these crustacean taxa, reproductive assurance in the face of fluctuating population sizes, habitat turnover or climate changes, appears to be a recurrent theme in the evolution of androdioecy. Flexibility in sexual system evolution has been maintained throughout the evolutionary history of Notostraca (over 250 my) and given the extreme morphological and life history conservatism in the group, could have facilitated their evolutionary persistence.

## Methods

### Sampling and sequencing

We produced de novo sequence data from 12 taxa from the two notostracan genera, *Triops* and *Lepidurus*[26]. Samples consisted of either sediments containing resting eggs or wild caught individuals preserved in 100% ethanol (Additional file 1: Table S1). Total genomic DNA was extracted from ethanol-preserved individuals using a Qiagen DNeasy Blood and Tissue Kit (Qiagen, Valencia, CA) or directly from individual resting eggs using a modified ‘HotSHOT’ procedure [90]. DNA sequences were generated for three mitochondrial gene fragments, COI, 12S rDNA and 16S rDNA, and four nuclear gene fragments; elongation factor 1 alpha, glycogen synthase, RNA polymerase II and 28S rDNA. We used primer pairs known to amplify across Notostraca for the mitochondrial and ribosomal genes [34,37,91] and designed new primers for the nuclear protein coding genes based on alignments of available sequences from notostracans and other branchiopods using PriFi [92] (see Additional file 1: Table S2 for primer sequences and optimised reaction conditions). Reactions were carried out in a final volume of 50 μl containing 2 μl of template DNA, 200 μM of each primer, 200 μM of each nucleotide, 0.01 U of *BioTaq* DNA polymerase (Bioline), 1x NH_4_ buffer (Bioline) and 2–3 mM MgCl_2_. Amplified fragments were purified and sequenced for both forward and reverse strands by Macrogen using an ABI 3730*xl* DNA Analyser (Macrogen Inc, Seoul, Korea). Sequences were manually edited using CodonCode Aligner v3.5 (CodonCode Corporation, Dedham, MA) with consensus sequences produced for each forward and reverse pair.

### GMYC model based species delimitation

Available Notostraca COI sequences were downloaded from GenBank (Additional file 1: Table S3) and aligned with our newly generated sequences in MEGA 5 [93] using MUSCLE [94] with default parameters. We applied a generalized mixed Yule coalescent (GMYC) model [95] to identify independently evolving clusters in our COI dataset, which correspond to STUs. First, we created an ultrametric phylogeny based on our COI alignment using BEAST v1.6.2 [96]. The phylogenetic analysis was run for 600,000 iterations with trees printed every 1,000 iterations and the first 100,000 iterations removed as burnin. A GTR + *Γ* nucleotide substitution model was used with a strict molecular clock with the rate fixed to 1. From this the ultrametric maximum clade credibility consensus tree was constructed. The GMYC analysis was performed in R v2.14.1 (R Development Core Team, 2011) with the package splits v1.0-11 (https://r-forge.r-project.org/projects/splits/). Clusters defined by the GMYC analysis were then assigned an STU I.D. based on the geographic location and species assignment of the accessions they contained. Uncorrected mean genetic distances in COI between STUs were calculated in MEGA 5 [93] with all positions containing gaps or missing data removed.

### Alignment and supermatrix construction

Single sequences for each gene for each STU identified were selected (where available) for inclusion in our multigene phylogenetic analysis (Additional file 1: Table S4). Sequences generated in this study were used preferentially but, where only GenBank sequences were available, records were checked to confirm that samples were from the same or close geographic location to samples used for STU identification.

The final alignment of each nuclear protein coding gene and the mitochondrial gene COI was carried out in MEGA 5 [93] with MUSCLE [94] using default parameters. The ribosomal genes were aligned based on secondary structure information using RNAsalsa v0.8.1 [97] with *Apis mellifera* structural data used as a constraint. Weakened constraint settings (S1, S2 and S3 = 0.51) were used to preserve structural information as described by Letsch and Kjer [98]. To confirm that the individual alignments were suitable for concatenation, phylogenetic congruence was tested with Concaterpillar v1.4 [99,100] using the GTR model and an α-level cut off of 0.05. No significant phylogenetic incongruence was identified (*p* = 0.55) and so all genes were concatenated using FASconCAT v1.0 [101]. The final supermatrix contained 5253 positions with 54% missing data. This number represents the overall missing data, not including indels, in the supermatrix alignment. It reflects the fact that most taxa retrieved from GenBank do not have coverage for all the genes used in this study and we could not obtain sequences for some genes for a few of our samples.

### Phylogenetic analysis

Phylogeny, based on the concatenated supermatrix, was inferred by ML and Bayesian Markov-chain Monte Carlo methods. We estimated the ML tree with RAxML using RAxMLHPC-PTHREADS v7.0.4 [100], treating each gene as an individual partition. An initial ML search using GTR + *Γ* was performed with 100 iterations to identify the best scoring ML tree. 1000 Bootstrap replicates were then conducted using GTR + *Γ* and drawn onto this best scoring ML tree. Bayesian phylogenetic analysis was conducted using BayesPhylogenies v1.0 [102] with a reversible jump mixture model [103] using a GTR model of sequence evolution with 4 discrete *Γ* rate categories. The analysis was run for 10,000,000 iterations with trees printed and saved every 10,000 iterations. Three independent rate matrices were assigned by BayesPhylogenies. Following this analysis the first 500,000 iterations were removed as burnin and the remaining 950 trees were used to create a consensus tree in BayesTrees v1.3 (http://www.evolution.rdg.ac.uk/BayesTrees.html).

### Sexual system

Male notostracans are readily identified by the lack of ovisacs, subtle morphological differences in carapace shape, numbers of legless rings and active mating behaviour in live individuals [26,32,48,56,104,105]. Females and hermaphrodites, although identical in external morphology and behaviour, differ histologically by the presence in hermaphrodites of an ovotestis (testicular lobes amongst the ovarian lobes) and by their ability to reproduce in isolation [40,50].

We compiled data from the literature for sex ratio, histology (i.e. presence/absence of ovotestis) and the inability/ability of females/hermaphrodites to reproduce in isolation (Table 1). Studies showing inability to reproduce in isolation were only included if reproduction in the presence of males was confirmed, to rule out poor lab rearing conditions or lack of reproductive maturity of individuals. In addition, we estimated sex ratio from available samples for a few populations (Additional file 1: Table S6). Using these data, we assigned populations as either being androdioecious or gonochoric. Androdioecious populations consist of hermaphrodites and males and exhibit skewed sex ratios with hermaphrodites found in greater numbers than males [12,17,48,82]. Gonochoric populations consist of males and females and have an approximately equal sex ratio. We did not categorise any population as purely hermaphroditic because this would necessitate showing a complete absence of males. Given that males in androdioecious species can be maintained by metapopulation dynamics [12,13,106] and can be present in exceedingly low frequencies (e.g. eight males per thousand in *L. apus* and similar proportions in *T. cancriformis*[52,53,107]) large samples sizes where no males are found would be needed to establish that a population is hermaphroditic [25]. In view of the sample sizes available to us we decided to conservatively categorise STUs into two sexual systems, gonochoric and androdioecious. In the AD notostracan species *T. newberryi* male proportions never exceed 27% [32,48] and in populations of AD *Eulimnadia* male proportions were always significantly lower than 50% male with a mode of ~20% [108]. Weeks et al. [108] did, however, note that upper values for population sex ratio of AD taxa overlapped with the lower values of gonochoric taxa in the 35% - 45% range. We therefore used a conservative population sex ratio cut-off of 30% male to assign an AD sexual system in the absence of additional histological or reproduction in isolation data in order to prevent misclassification due to stochastic variation in natural population sex ratios [51,109]. Populations with a male proportion of 30% - 45% were coded as equivocal and populations with male proportions greater than 45% were coded as gonochoric.

### Character mapping

Sexual system was mapped onto the best scoring ML tree as a discrete character. STUs for which sexual system could not be inferred, or for which data was lacking, were left uncoded for sexual system in our analyses. The *Leptestheria* outgroup used to root the tree for character mapping analyses was also left uncoded for sexual system. MP reconstruction of ancestral states was conducted using Mesquite v2.74 [110] with an unordered model. In addition, we used BayesMultistate [111] implemented in BayesTraits v1.0 in an ML framework to evaluate four alternative models of sexual system evolution using likelihood ratio tests (*D*) assuming the result approximates a chi-squared distribution with degrees of freedom equal to the difference in the number of estimated parameters between the models. The simplest model is a one-parameter model, with a single rate of transition between gonochorism and AD and vice versa. The second model is a two-parameter model, where the transition rates from AD to gonochorism and vice versa can vary. The third and fourth models allow only unidirectional changes in sexual system, one from gonochorism to AD only, as in clam shrimps [25], and the other from AD to gonochorism only. Ancestral character states were reconstructed based on the best fit model using the AddNode function of BayesTraits.

### Testing the reproductive assurance hypothesis

We used a proxy for the exposure of STUs to glacial cycles, and therefore presumed range expansions, to test whether the reproductive assurance hypothesis is responsible for sexual system evolution in Notostraca [39,40]. As STUs found at higher latitudes are more likely to have recently re-colonised following the last glacial maxima than lower latitude ones, we expect AD STUs to be found at higher latitudes than gonochoric ones. The absolute latitude values at which gonochoric and AD STUs are found were compiled using the collection location of each representative STU as an unbiased representation of the latitude at which that lineage is found (Additional file 1: Table S5). We used the program BayesTraits [111] in an ML framework to conduct a *t*-test which accounts for the shared ancestry as implied by our best scoring ML phylogeny (phylogenetic *t*-test) to determine if latitude significantly differs between gonochoric STUs and ones where AD populations are found (the presence and absence of AD was incorporated using standard contrast or ‘dummy’ coding). We simultaneously estimated the parameter λ which detects the phylogenetic signal in the data [112,113], if λ is close to 1 there is strong phylogenetic signal if it is 0 there is no phylogenetic signal and the model collapses to an ordinary *t*-test.

## Availability of Supporting Data

Supermatrix alignment used for phylogenetic inference has been deposited in the Dryad repository under http://dx.doi.org/10.5061/dryad.480cf.

## Competing interests

The authors declare that they have no competing interest.

## Authors’ contributions

TCM collected and analysed data and drafted the manuscript. AG, BH, RAJ and RH conceived the study. TZ collected some samples and provided some sequences. All authors read, contributed to and approved the final manuscript.

## Supplementary Material

Additional file 1 **Mathers et al.** High lability of sexual system over 250 million years of evolution in morphologically conservative tadpole shrimps. Click here for file
