# Supplementary material for: High lability of sexual system over 250 million years of evolution in morphologically conservative tadpole shrimps
Source: BMC Evol Biol. 2013 Feb 5;13:30. doi: 10.1186/1471-2148-13-30 (PMC3585860; doi:10.1186/1471-2148-13-30)
Supplement: Additional file 1 — Mathers et al. High lability of sexual system over 250 million years of evolution in morphologically conservative tadpole shrimps. [file 1471-2148-13-30-S1.docx]

Additional File 1

**Mathers et al.: High lability of sexual system over 250 million years of evolution in morphologically conservative tadpole shrimps.**

**Additional methods**

**Relaxed clock phylogenetic analysis**

BEAST v1.7.4 [[1](#_ENREF_1)] was used to create two ultrametric phylogenies; one based on the full dataset and a second using a reduced dataset containing only the mitochondrial (mt) genes 12S, 16S and COI. We used an uncorrelated log normal relaxed clock and Yule speciation prior for both analyses. As with the unconstrained ML and Bayesian phylogenetic analyses each gene was treated as a separate partition with substitution model parameters estimated independently (unlinked). All partitions were initially assigned a GTR + gamma model of sequence evolution. However, test runs showed poor mixing of some GTR model parameters for the 12S, 16S, 28S and RNA polymerase II partitions. We therefore simplified the substitution model to HKY + gamma for these partitions (12S and 16S only in the mt analysis). Two independent MCMC chains were run for 100 million iterations, sampling every 10,000 iterations, for each analysis. Runs were checked for convergence and adequate mixing using Tracer v1.5. ESS values were greater than 1000 for all parameters in all runs. A posterior sample of 8,000 trees from one of the runs for each analysis was used to create a maximum clade credibility tree with TreeAnotator (http://beast.bio.ed.ac.uk/TreeAnnotator) (see figures S2 and S3). These two trees were then used to run the ML character mapping analysis in BayesTraits (Table S7).

**Table S1**: Details of samples collected for this study with sample type, location, coordinates, infered STU following the GMYC barcoding analysis and details of genes sequenced with accession numbers for each sample. For sample type GB = GenBank data, RE = resting egg, LR = lab reared, WC = wild caught.

|  |  | | | | | **Genes Sequenced with accession numbers** | | | | | | |
| --- | --- | --- | --- | --- | --- | --- | --- | --- | --- | --- | --- | --- |
| **Sample I.D.** | | **Sample type** | **Location** | **Coordinates** | **STU I.D. (From GMYC)** | **COI** | **12S** | **16S** | **28S** | **EF1-α** | **RNA PII** | **Glyc. Synth.** |
| T_m_D2 | | LR | Doñana national park, Spain |  | *Triops baeticus* | Y | Y | Y |  | Y | Y |  |
| T_c_ESP | | LR | Espolla, Spain | 42°09'02" N / 002°45'60" E | *Triops cancriformis* | Y | Y | Y | Y | Y | Y | Y |
| T_g_CC | | RE | Cornish Crispa Co. commercial kit, “Triops granarius” |  | *Triops granarius* (Japan) | Y | Y | Y | Y | Y | Y |  |
| T_g_Namibia | | WC | Arandis, Namibia | 22°24'27'' S / 14°58'24'' E | *Triops granarius* (Namibia) | Y |  |  |  |  |  |  |
| T_long_CW | | LR | Clearwater, Kansas, USA |  | *T. cf. longicaudatus sp. 2* | Y | Y | Y |  | Y | Y |  |
| T_bb | | LR | Corni Crispa Co. Comercial kit, “Black Beauty Triops” |  | *T. cf. longicaudatus sp. 2* | Y |  |  |  |  |  |  |
| T_n | | RE | Shallow Water, Kansas, USA |  | *Triops newberryi* | Y | Y | Y | Y | Y | Y | Y |
| L_apus | | WC | Frankfurt-Oder, Germany | 52°19'29" N / 14°33'33" E | *Lepidurus apus* | Y |  |  | Y |  |  |  |
| L_arc | | WC | Strandvolldammane, Svalbard | 78.96061 N / 11.50817 E | *Lepidurus arcticus* | Y |  |  |  | Y | Y | Y |
| Ll_MS | | WC | Castel Porziano Estate, Italy | 41.74748 N / 12.41860 E | *Lepidurus lubbocki* | Y | Y | Y | Y |  | Y |  |
| L_pack | | LR | Jeppsen Prarie Reserve, California |  | *Lepidurus packardi* | Y | Y | Y | Y | Y |  |  |
| L_sp_Sardinia | | WC | Giara di Gesturi, Sardinia | 39°44'30" N / 8°59'51" E | *Lepidurus sp.* (Sardinia) | Y | Y | Y | Y | Y | Y | Y |

Table S2: Primers and PCR conditions used for the amplification and sequencing of mitochondrial and nuclear markers. A general PCR program was used with annealing temperatures given below: 94 ºC 2 min, (94 ºC 30 s., annealing temp. 30 s. 72 ºC 1 min) x 35, 72 ºC 5 min.

| **Gene** | **Name** | **Primer sequence (5’->3’)** | **Annealing temp. (ºC)** | **MgCL_2_ conc. (mM)** | **Reference** |
| --- | --- | --- | --- | --- | --- |
| 12S | 12S_col_F  12S_col_R | ATGCACTTTCCAGTACATCTAC  AAATCGTGCCAGCCGTCGC | 50 | 1 | [[2](#_ENREF_2)] |
| 16S | 16Sar  16Sbr | CGCCTGTTTATCAAAAACAT  CCGGTCTGAACTCAGATCACGT | 50 | 1 | [[3](#_ENREF_3)] |
| 28S | 28S_L1  28S_H4lv1 | AGCGGAGGAAAAGAAACTA  ACGATCGATTTGCACGTCAG | 58 | 2 | [[4](#_ENREF_4)] |
| COI | LCO1490  HC02198 | GGTCAACAAATCATAAAGATATTGG  TAAACTTCAGGGTGACCAAAAAATCA | 52 | 2 | [[5](#_ENREF_5)] |
| Elongation factor 1 α | EF1_F  EF1_R | TCGAAACCGCCAAGTTCTAC  GTAACCACGACGCAATTCCT | Touch down 55 – 45* | 3 | This Study |
| Glycogen synthase | Glyc_F  Glyc_R | CGACGAAGAAGCTGGAAAAC  GACAAAAACTCGGGATGGAA | Touch down 55 – 45* | 1.5 | This Study |
| RNA polymerase II | RNA_P_F  RNA_P_R | GGATTTCTCCWGGAGATACSAAGGTCATGG  TCGACGTTCTGTTGACCKACGCAAGC | Touch down 55 – 45* | 2 | This Study |

* -0.4 per cycle for 25 cycles, then 10 cycles at 45ºC.

Table S3: Results of the STU cluster analysis on Notostraca COI sequences. Accession numbers of Notostraca COI sequences used in the GMYC analysis are provided, as well as the reference for the STU ID. New accessions for this study are in bold.

| **STU/Cluster ID** | **Accession** | **Reference** |
| --- | --- | --- |
| *L. apus* | DQ834543.1 | [[6](#_ENREF_6), [7](#_ENREF_7)] |
|  | DQ834544.1 |  |
|  | DQ148285.1 |  |
|  | EF189669.1 |  |
|  | **JX110638** |  |
| *L. arcticus* | HM425362.1 BOLD | [[8](#_ENREF_8)] |
|  | AF209067.1 |  |
|  | DQ834545.1 |  |
|  | HM425363.1 BOLD |  |
|  | HM425361.1 BOLD |  |
|  | DQ148286.1 |  |
|  | HM425364.1 BOLD |  |
|  | HM425365.1 BOLD |  |
|  | **JX110641** |  |
| *L. cf. couessi* (Italy) | DQ148290.1 | [[7](#_ENREF_7)] |
|  | DQ148288.1 |  |
|  | DQ834549.1 |  |
|  | DQ834546.1 |  |
|  | DQ148289.1 |  |
|  | DQ834547.1 |  |
|  | DQ834548.1 |  |
|  | DQ834550.1 |  |
|  | DQ148287.1 |  |
| *L. couessi* | DQ310622.1 | [[8](#_ENREF_8)] |
|  | DQ889156.1 |  |
| *L. lemmoni* | GQ144447.1 | [[8](#_ENREF_8)] |
| *L. lubbocki* | DQ148284.1 | [[7](#_ENREF_7)] |
|  | DQ834542.1 |  |
|  | **JX110643** |  |
|  | DQ148283.1 |  |
|  | DQ148282.1 |  |
|  | DQ834541.1 |  |
|  | DQ834540.1 |  |
| *L. packardi* | **JX110642** | [[8](#_ENREF_8)] |
|  |  |  |
| *L. cf. couesii* (Sardinia) | **JX110640** |  |
| *T. baeticus* | FN691434.1 | [[9](#_ENREF_9)] |
|  | **JX110645** |  |
|  | FN691433.1 |  |
| *T. cancriformis* | EF675884.1 | [[10](#_ENREF_10)] |
|  | EF675896.1 |  |
|  | EF675892.1 |  |
|  | EF675885.1 |  |
|  | EF675895.1 |  |
|  | EF675887.1 |  |
|  | EF675888.1 |  |
|  | EF675893.1 |  |
|  | EF675886.1 |  |
|  | EF675891.1 |  |
|  | EF675894.1 |  |
|  | EF675897.1 |  |
|  | EF675889.1 |  |
|  | EF675890.1 |  |
|  | FN691430.1 |  |
|  | EF675899.1 |  |
|  | EF675898.1 |  |
|  | GQ328960.1 |  |
|  | GQ144445.1 |  |
|  | FN691431.1 |  |
|  | DQ369312.1 |  |
|  | EF675859.1 |  |
|  | EF675860.1 |  |
|  | EF675853.1 |  |
|  | EF675832.1 |  |
|  | EF675842.1 |  |
|  | EF675843.1 |  |
|  | EF675844.1 |  |
|  | EF675850.1 |  |
|  | EF675845.1 |  |
|  | EF675831.1 |  |
|  | EF675836.1 |  |
|  | EF675835.1 |  |
|  | EF675879.1 |  |
|  | EF675851.1 |  |
|  | EF675829.1 |  |
|  | EF675849.1 |  |
|  | EF675838.1 |  |
|  | EF675837.1 |  |
|  | EF675840.1 |  |
|  | EF675861.1 |  |
|  | EF675857.1 |  |
|  | EF675830.1 |  |
|  | EF675848.1 |  |
|  | EF675854.1 |  |
|  | EF675856.1 |  |
|  | EF675852.1 |  |
|  | EF675834.1 |  |
|  | EF675846.1 |  |
|  | EF675839.1 |  |
|  | EF675833.1 |  |
|  | EF675847.1 |  |
|  | EF675841.1 |  |
|  | EF675858.1 |  |
|  | EF675855.1 |  |
|  | DQ369315.1 |  |
|  | **JX110644** |  |
|  | EF675869.1 |  |
|  | EF675872.1 |  |
|  | EF675866.1 |  |
|  | EF675868.1 |  |
|  | EF675878.1 |  |
|  | DQ369317.1 |  |
|  | EF675874.1 |  |
|  | EF675875.1 |  |
|  | EF675870.1 |  |
|  | EF675864.1 |  |
|  | EF675867.1 |  |
|  | EF675873.1 |  |
|  | EF675863.1 |  |
|  | EF675871.1 |  |
|  | EF675876.1 |  |
|  | EF675865.1 |  |
|  | EF675877.1 |  |
|  | EF675862.1 |  |
|  | DQ369314.1 |  |
|  | EF675827.1 |  |
|  | DQ369313.1 |  |
|  | FN691432.1 |  |
|  | EF675826.1 |  |
|  | NC 004465.1 |  |
|  | EF675828.1 |  |
|  | EF189678.1 |  |
|  | AB084514.1 |  |
|  | DQ148291.1 |  |
|  | DQ664196.1 |  |
|  | EF675880.1 |  |
|  | EF675881.1 |  |
|  | DQ369316.1 |  |
|  | EF675882.1 |  |
|  | EF675883.1 |  |
| *T. cf. australiensis sp. 1* | EF189677.1 | This study |
| *T. cf. australiensis sp. 2* | DQ310624.1 | This study |
|  | DQ889135.1 |  |
| *T. cf. australiensis sp. 3* | DQ310625.1 | This study |
| *T. cf. australiensis sp. A* | DQ343234.1 | [[11](#_ENREF_11)] |
| *T. cf. australiensis sp. B* | DQ343235.1 | [[11](#_ENREF_11)] |
| *T. cf. longicaudatus sp. 1* | HQ908557.1 | [[12](#_ENREF_12)] |
|  | HQ908544.1 |  |
|  | HQ908563.1 |  |
|  | HQ908559.1 |  |
|  | HQ908554.1 |  |
|  | HQ908552.1 |  |
|  | HQ908548.1 |  |
|  | HQ908547.1 |  |
|  | HQ908550.1 |  |
|  | HQ908567.1 |  |
|  | HQ908564.1 |  |
|  | HQ908551.1 |  |
|  | HQ908556.1 |  |
|  | HQ908546.1 |  |
|  | HQ908565.1 |  |
|  | HQ908558.1 |  |
|  | HQ908561.1 |  |
|  | HQ908549.1 |  |
|  | HQ908566.1 |  |
|  | HQ908555.1 |  |
|  | HQ908560.1 |  |
|  | HQ908553.1 |  |
|  | HQ908545.1 |  |
|  | HQ908562.1 |  |
| *T. emeritensis* | EF675900.1 | [[9](#_ENREF_9)] |
|  | FN691435.1 |  |
| *T. cf. granarius* (Japan) | GQ144446.1 | [[4](#_ENREF_4)] |
|  | **JX110646** |  |
| *T. cf. granarius* (Namibia) | **JX110639** | [[4](#_ENREF_4)] |
| *T. cf. longicaudatus sp. 2* | HQ908538.1 | This study / [[12](#_ENREF_12)] |
|  | HQ908539.1 |  |
|  | HQ908526.1 |  |
|  | HQ908523.1 |  |
|  | HQ908542.1 |  |
|  | HQ908531.1 |  |
|  | HQ908524.1 |  |
|  | HQ908530.1 |  |
|  | **JX110649** |  |
|  | HQ908519.1 |  |
|  | HQ908527.1 |  |
|  | HQ908525.1 |  |
|  | HQ908521.1 |  |
|  | HQ908534.1 |  |
|  | HQ908540.1 |  |
|  | HQ908541.1 |  |
|  | HQ908518.1 |  |
|  | HQ908522.1 |  |
|  | HQ908543.1 |  |
|  | HQ908537.1 |  |
|  | HQ908536.1 |  |
|  | HQ908533.1 |  |
|  | HQ908535.1 |  |
|  | HQ908529.1 |  |
|  | HQ908520.1 |  |
|  | HQ908517.1 |  |
|  | HQ908532.1 |  |
|  | HQ908528.1 |  |
|  | GU475465.1 |  |
|  | HM883938.1 BOLD |  |
|  | HM883939.1 BOLD |  |
|  | HM883941.1 BOLD |  |
|  | HM883940.1 BOLD |  |
|  | HM883942.1 BOLD |  |
|  | GQ144444.1 |  |
|  | DQ310623.1 |  |
|  | **JX110647** |  |
| *T. mauritanicus* | EF675905.1 | [[9](#_ENREF_9), [10](#_ENREF_10)] |
|  | EF675904.1 |  |
|  | EF675901.1 |  |
|  | EF675903.1 |  |
|  | EF675902.1 |  |
|  | FN691439.1 |  |
|  | FN691440.1 |  |
|  | FN691443.1 |  |
|  | FN691442.1 |  |
|  | FN691441.1 |  |
| *T. newberryi* | HQ908510.1 | [[12](#_ENREF_12)] |
|  | HQ908508.1 |  |
|  | HQ908509.1 |  |
|  | HQ908496.1 |  |
|  | HQ908507.1 |  |
|  | HQ908499.1 |  |
|  | HQ908501.1 |  |
|  | HQ908502.1 |  |
|  | HQ908512.1 |  |
|  | HQ908500.1 |  |
|  | HQ908498.1 |  |
|  | HQ908504.1 |  |
|  | HQ908505.1 |  |
|  | HQ908506.1 |  |
|  | HQ908503.1 |  |
|  | HQ908511.1 |  |
|  | HQ908513.1 |  |
|  | HQ908516.1 |  |
|  | HQ908514.1 |  |
|  | HQ908497.1 |  |
|  | **JX110648** |  |
|  | HQ908515.1 |  |
|  | NC 006079.1 |  |
|  | AY639934.1 |  |
| *T. simplex* | FN691438.1 | [[9](#_ENREF_9)] |
|  | FN691436.1 |  |
|  | FN691437.1 |  |
| *T. cf. mauritanicus* (Ares) | EF675906.1 | This study |
|  | EF675907.1 |  |
|  | EF675908.1 |  |
| *T. cf. granarius* (Russia) | FN691444.1 | This study |
| *T. vicentinus* | FN691444.1 | [[9](#_ENREF_9)] |

Table S4: Samples and accession numbers of the sequences used for phylogenetic analysis. GB = GenBank data, RE = resting egg, LR = lab reared, WC = wild caught. Newly generated sequences are shown in bold.

|  | **Genetic data used with accession number** | | | | | | | |
| --- | --- | --- | --- | --- | --- | --- | --- | --- |
| **STU** | **12S** | **16S** | **COI** | **28S** | **EF1α** | **RNA PII** | **Glyc. Synth** |  |
| *T. baeticus* | **KC466334** | **KC466343** | **JX110645** | - | **KC466360** | **KC466372** | - |  |
| *T. cancriformis* | **KC466333** | **KC466342** | **JX110644** | **KC466348** | **KC466359** | **KC466368** | **KC466363** |  |
| *T. cf. australiensis sp. 1* | - | EF189616 | EF189677 | EF189662 | EF189595 | - | - |  |
| *T. cf. australiensis sp. 2* | - | - | DQ310624 | - | - | - | - |  |
| *T. cf. australiensis sp. 3* | - | - | DQ310625 | - | - | - | - |  |
| *T. cf. australiensis sp. A* | DQ343232 | - | DQ343234 | - | - | - | - |  |
| *T. cf. australiensis sp. B* | DQ343233 | - | DQ343235 | - | - | - | - |  |
| *T. cf. granarius* (Japan) | **KC466335** | **KC466341** | **JX110646** | **KC466347** | **KC466358** | **KC466369** | - |  |
| *T. cf. granarius* (Namibia) | AM269423 | AM269433 | **JX110639** | AM269444 | - | - | - |  |
| *T. cf. granarius* (Tunisia) | AM269421 | AM269431 | - | AM269442 | - | - | - |  |
| *T. cf. longicaudatus sp. 1* | - | - | HQ908544 | - | - | - | - |  |
| *T. cf. longicaudatus sp.2* | **KC466336** | **KC466344** | **JX110649** | - | **KC466356** | **KC466371** | - |  |
| *T. emeritensis* | FN691428 | AM183882 | FN691435 | - | - | - | - |  |
| *T. gadensis* | FN691421 | FN689863 | - | - | - | - | - |  |
| *T. mauritanicus* | AM184177 | AM183873 | FN691439 | - | - | - | - |  |
| *T. newberryi* | **KC466337** | **KC466345** | **JX110648** | **KC466346** | **KC466357** | **KC466370** | **KC466364** |  |
| *T. simplex* | AM184172 | AM183867 | FN691436 | - | - | - | - |  |
| *T. cf. mauritanicus* (E Spain) | - | - | EF675907 | - | - | - | - |  |
| *T. cf. granarius* (Russia) | - | - | EF521890 | - | - | - | - |  |
| *T. vicentinus* | FN691426 | FN689867 | FN691444 | - | - | - | - |  |
| *L. apus* | AF494483 | DQ148279 | **JX110638** | **KC466349** | AF526293 | - | - |  |
| *L. arcticus* | AY159569 / AJ583699 * | DQ834538 | **JX110641** | AF209047 | **KC466353** | **KC466365** | **KC466361** |  |
| *L. bilobatus* | AJ000828 | - | - | - | - | - | - |  |
| *L. couesii* (Canada) | AJ000827 | - | DQ310622 | - | - | - | - |  |
| *L. cf. couesii* (Italy) | DQ148274 | DQ148280 | DQ834546 | - | - | - | - |  |
| *L. cf. couesii* (Sardinia) | **KC466331** | **KC466339** | **JX110640** | **KC466351** | **KC466355** | **KC466366** | **KC466362** |  |
| *L. cryptus* | AJ000824 | - | - | - | - | - | - |  |
| *L. lemmoni* | AY115604 | AY115614 | GQ144447 | - | - | - | - |  |
| *L. lubbocki* | **KC466332** | **KC466340** | **JX110643** | **KC466350** | - | **KC466367** | - |  |
| *L. packardi* | **KC466330** | **KC466338** | **JX110642** | **KC466352** | **KC466354** | - | - |  |
| *Leptestheria sp.*(Outgroup) | DQ872782 | EF189606 | DQ872786 | - | FJ499045 | - | - |  |

*two concatenated sequences were used for this gene fragment.

Table S5: Latitude data for taxa with known reproductive mode

| **STU** | **AD** | **Latitude** |
| --- | --- | --- |
| *T. baeticus* | No | 36 |
| *T. cancriformis* | Yes | 42 |
| *T. cf. australiensis sp. A* | No | 29 |
| *T. cf. australiensis sp. B* | Yes | 29 |
| *T. cf. granarius* (Japan) | No | 36 |
| *T. cf. granarius* (Namibia) | No | 22 |
| *T. cf. granarius* (Tunisia) | No | 35 |
| *T. cf. longicaudatus sp.2* | Yes | 37 |
| *T. cf. longicaudatus sp. 1* | No | 32 |
| *T. emeritensis* | No | 39 |
| *T. newberryi* | Yes | 38 |
| *T. simplex* | No | 36 |
| *T. vicentinus* | No | 37 |
| *L. apus* | Yes | 52 |
| *L. arcticus* | Yes | 78 |
| *L. cf. couesii* (Sardinia) | No | 39 |
| *L. couesii* (Canada) | No | 53 |
| *L. lemmoni* | No | 34 |
| *L. lubbocki* | No | 41 |
| *L. packardi* | No | 38 |

**Table S6:** Notostraca reproductive data. STU I.D., population locations, the presence of genetic barcoding data (COI, 12S or 16S) to support species assignment, sex ratio (percent male), and sample sizes (where available), histology data (presence of ovotestis in individuals) and the inferred sexual system used in the character evolution analysis is given. Where genetic data for a population is published in a separate study to the sexual system data both references are given.

| **Significant Taxonomic Units (STU)** | **Location** | **Genetic barcode for population** | **Sex ratio** | **N** | **Reproduction in isolation** | **Ovotestis** | **Sexual system** | **References** |
| --- | --- | --- | --- | --- | --- | --- | --- | --- |
| *T. baeticus* | Southern Iberia – Multiple populations | Y | 51 | 255 |  |  | Gonochoric | [[9](#_ENREF_9)] |
| *T. cancriformis* | Across Europe | Y | 0 -53 |  | Yes^1^ | Yes ^1^ | Gonochoric / AD | [[13-17](#_ENREF_13)] |
| *T. cf. australiensis sp. 1* |  | - |  |  |  |  | No data |  |
| *T. cf. australiensis sp. 2* |  | - |  |  |  |  | No data |  |
| *T. cf. australiensis sp. 3* |  | - |  |  |  |  | No data |  |
| *T. cf. australiensis sp. A* | Bourke, Paroo, Australia | Y | 47 | 15 |  | No | Gonochoric | [[11](#_ENREF_11)] |
| *T. cf. australiensis sp. B* | Bourke, Paroo, Australia | Y | 0 | 22 |  | Yes | AD | [[11](#_ENREF_11)] |
| *T. emeritensis* | Badajoz, Spain – Multiple populations | Y | 55 | 29 |  |  | Gonochoric | [[9](#_ENREF_9)] |
| *T. gadensis* | Cadiz, Spain – Multiple populations | Y | 36 | 52 |  |  | Equivocal | [[9](#_ENREF_9)] |
| *T. cf. granarius* (Japan) | 3 locations: Shizuoka, Kagawa, Fukuoka | Y | 49 | 388 | No |  | Gonochoric | [[15](#_ENREF_15), [16](#_ENREF_16), [18](#_ENREF_18), [19](#_ENREF_19)] |
| *T. cf. granarius* (Namibia) |  | Y | Even^2^ |  | No |  | Gonochoric | [[4](#_ENREF_4), [15](#_ENREF_15), [16](#_ENREF_16)] |
| *T. cf. granarius* (Tunisia) |  | Y | Even^2^ |  | No |  | Gonochoric | [[4](#_ENREF_4), [15](#_ENREF_15), [16](#_ENREF_16)] |
| *T. cf. longicaudatus sp. 1* (Long bodied - Macdonald) | Northern Chihuahuan desert, USA | Y | 48 | 21 |  |  | Gonochoric | [[12](#_ENREF_12)] |
| *T. cf. longicaudatus sp. 2* | Zacatecas, Mexico | Y | 53 | 108 |  | No | Gonochoric | [[20](#_ENREF_20), [21](#_ENREF_21)] |
| *T. cf. longicaudatus sp. 2* | Clearwater, Kansas | Y | 50 | 6 |  |  | Gonochoric | Own data |
|  |  |  |  |  |  |  |  |  |
| *T. cf. longicaudatus sp. 2* | Northern Chihuahuan desert, USA | Y | 0 | 212 |  |  | AD | [[12](#_ENREF_12)] |
| *T. newberryi* | Multiple populations from Northern Chihuahuan desert, USA | Y | 0 - 27 | 427 |  |  | AD | [[12](#_ENREF_12)] |
| *T. newberryi* | Multiple populations from California, Nevada and Utah | N | 0 – 16 | 487 | Yes |  | AD | [[22](#_ENREF_22)] |
| *T. mauritanicus* |  | - |  |  |  |  | No data |  |
| *T. simplex* | Kairouan, Tunisia | Y | 53 | 17 |  |  | Gonochoric | [[10](#_ENREF_10)] |
| *T. vicentinus* | SW Portugal – Multiple populations | Y | 50 | 70 |  |  | Gonochoric | [[9](#_ENREF_9)] |
| *T. vicentinus* | Vila do Bispo, SW Portugal, 2 populations | Y | 58 | 118 |  |  | Gonochoric | [[23](#_ENREF_23)] |
| *L. apus* | Frankfurt, Germany | Y | 0 | 62 |  |  | AD | Own data |
| *L. apus* | Germany | N | 5 | 411 |  |  | AD | [[24](#_ENREF_24)] |
| *L. apus* | Gosseberg, Austria | N | 1 | 2712 |  |  | AD | [[25](#_ENREF_25)] |
| *L. apus* | Ivry, France | N | 18 | 174 |  |  | AD | [[26](#_ENREF_26)] |
| *L. arcticus* | Rasshua,Kuril Islands, Russia |  | 7 | 60 |  |  | AD |  |
| *L. arcticus* | Cambridge Bay, Canada | N | 7 | 14 |  |  | AD | [[27](#_ENREF_27)] |
| *L. arcticus* | Ooglamie, Alaska | N | 0 | 113 |  |  | AD | [[27](#_ENREF_27)] |
| *L. arcticus* | Broughton Island, Canada | N | 1 | 109 |  |  | AD | [[28](#_ENREF_28)] |
| *L. arcticus* | Spitsbergen, Svalbard | N | 0 | 200 |  |  | AD | [[29](#_ENREF_29)] |
| *L. bilobatus* | Irish Canyon, Moffat, Colorado, USA | Y | 35 | 116 |  |  | Equivocal | [[30](#_ENREF_30), [31](#_ENREF_31)] |
| *L. couesii* | Frenchman River, Montana, USA | N | 51 | 63 |  |  | Gonochoric | [[32](#_ENREF_32)] |
| *L. cf. couesii* (Italy) |  | - |  |  |  |  | No data | - |
| *L. cryptus* |  | - |  |  |  |  | No data | - |
| *L. lemmoni* | Grand Couleee, Washington State, USA | N | 56 | 96 |  |  | Gonochoric | [[27](#_ENREF_27)] |
| *L. lemmoni* | Lassen County, California, USA | N | Even^2^ | 247 |  |  | Gonochoric | [[33](#_ENREF_33)] |
| *L. lubbocki* | Israel, 9 populations | N | Median = 54 |  |  |  | Gonochoric | [[34](#_ENREF_34)] |
| *L. lubbocki* | Castel Porziano Estate, Italy | Y | Even ^2^ |  |  | No | Gonochoric | [[6](#_ENREF_6), [7](#_ENREF_7), [35](#_ENREF_35)] |
| *L. packardi* | Tehama County, California | N | ~ 50 (over several months) |  |  |  | Gonochoric | [[36](#_ENREF_36)] |
| *L. cf. couesii* (Sardinia) | Giara di Gesturi, Sardinia | Y | 54 | 208 |  |  | Gonochoric | [[37](#_ENREF_37)] |

^1^ Not in populations that have equal sex ratio.

^2^ Reported gonochoric with even sex ratio, exact numbers not given.

**Table S6:** Comparison of BayesTraits character mapping results using three alternative phylogenetic trees – an ML unconstrained phylogeny using the full dataset, an ultrametric phylogeny created with BEAST under a lognormal relaxed molecular clock based on the full dataset and an ultrametric phylogeny created with BEAST under a lognormal relaxed molecular clock based on a reduced dataset containing only the mitochondrial genes 12S, 16S and COI. Under each phylogeny, models of transitions in sexual system were compared using the ML implementation of BayesMultistate. lnL = log-likelihood of model, *q_GA_* = transition rate from gonochorism to androdioecy, *q_AG_* = transition rate from androdioecy to gonochorism, *p* = *p*-value for *D* with 1 degree of freedom comparing the restricted models to the unrestricted model. The best fitting model under each phylogeny is highlighted in green.

| **Phylogenetic tree used** | **Model** | **lnL** | ***q_GA_*** | ***q_AG_*** | ***p*** |
| --- | --- | --- | --- | --- | --- |
| Unconstrained ML (RAxML), full dataset | Unrestricted | -7.229 | 23.729 | 110.640 | - |
|  | Equal rates | -10.262 | 5.730 | 5.730 | 0.0138 |
|  | AD to gonochorism only | -16.855 | 0.000 | 6.673 | < 0.0001 |
|  | Gonochorism to AD only | -12.0864 | 1.755 | 0.000 | 0.0018 |
| Ultrametric (BEAST), lognormal relaxed molecular clock, full dataset | Unrestricted | -8.841 | 25.144 | 87,939 | - |
|  | Equal rates | -11.013 | 6.776 | 6.776 | 0.0371 |
|  | AD to gonochorism only | -13.715 | 0.000 | 8.686 | 0.0018 |
|  | Gonochorism to AD only | -12.334 | 2.681 | 0.000 | 0.0082 |
| Ultrametric (BEAST), lognormal relaxed molecular clock, Mt only | Unrestricted | -8.841 | 133.513 | 467.296 | - |
|  | Equal rates | -10.975 | 2.036 | 2.036 | 0.0389 |
|  | AD to gonochorism only | -13.340 | 0 | 6.395 | 0.0027 |
|  | Gonochorism to AD only | -12.209 | 1.975 | 0 | 0.0095 |
|  |  |  |  |  |  |

Figure S1: Ultrametric consensus phylogeny created in BEAST based on aligned COI sequences from GenBank and our newly generated sequence data showing collapsed clusters identified by GMYC analysis. Accessions within each cluster are listed in Table S1.

**Figure S2:** Ultrametric BEAST maximum clade credibility tree based on the full dataset. Values at nodes show posterior probabilities.

**Figure S3:** Ultrametric BEAST maximum clade credibility tree based on the full dataset. Values at nodes show posterior probabilities.

**References**

1. Drummond AJ, Suchard MA, Xie D, Rambaut A: **Bayesian phylogenetics with BEAUti and the BEAST 1.7.** *Molecular Biology and Evolution* 2012.

2. Colbourne JK, Hebert PDN: **The systematics of North American Daphnia (Crustacea: Anomopoda): A molecular phylogenetic approach.** *Philosophical Transactions of the Royal Society of London Series B-Biological Sciences* 1996, **351:**349-360.

3. Palumbi S, Martin A, Romano S, McMillan W, Stice L, Grabowski G: **The simple fool’s guide to PCR, Version 2. .** *Special Publication of Department of Zoology, University of Hawaii, Honolulu, Hawaii, USA* 1991**:**28.

4. Korn M, Hundsdoerfer AK: **Evidence for cryptic species in the tadpole shrimp *Triops granarius* (Lucas, 1864) (Crustacea : Notostraca).** *Zootaxa* 2006, **1257:**57-68.

5. Folmer O, Black M, Hoeh W, Lutz R, Vrijenhoek R: **DNA primers for amplification of mitochondrial cytochrome c oxidase subunit I from diverse metazoan invertebrates.** *Molecular Marine Biology and Biotechnology* 1994, **3:**294-299.

6. Mantovani B, Cesari M, Scanabissi F: **Molecular taxonomy and phylogeny of the 'living fossil' lineages *Triops* and *Lepidurus* (Branchiopoda : Notostraca).** *Zoologica Scripta* 2004, **33:**367-374.

7. Mantovani B, Cesari M, Scanabissi F: **Molecular taxonomy and phylogeny of Italian *Lepidurus* taxa (Branchiopoda: Notostraca).** *Italian Journal of Zoology* 2009, **76:**358-365.

8. Rogers DC: **Revision of the nearctic Lepidurus (Notostraca).** *Journal of Crustacean Biology* 2001, **21:**991-1006.

9. Korn M, Green AJ, Machado M, Garcia-de-Lomas J, Cristo M, da Fonseca LC, Frisch D, Perez-Bote JL, Hundsdoerfer AK: **Phylogeny, molecular ecology and taxonomy of southern Iberian lineages of *Triops mauritanicus* (Crustacea: Notostraca).** *Organisms Diversity and Evolution* 2010, **10:**409-440.

10. Korn M, Marrone F, Perez-Bote JL, Machado M, Cristo M, da Fonseca LC, Hundsdoerfer AK: **Sister species within the *Triops cancriformis* lineage (Crustacea, Notostraca).** *Zoologica Scripta* 2006, **35:**301-322.

11. Murugan G, Obregon-Barboza H, Maeda-Martinez AM, Timms BV: **Co-occurrence of two tadpole shrimp, *Triops cf. australiensis* (Branchiopoda : Notostraca), lineages in middle Paroo, north-western New South Wales, with the first record of *Triops* hermaphrodites for the Australian continent.** *Australian Journal of Zoology* 2009, **57:**77-84.

12. Macdonald KS, Sallenave R, Cowley DE: **Morphological and genetic variation in *Triops* (Branchiopoda: Notostraca) from ephemeral waters of the Northern Chihuahuan desert of North America.** *Journal of Crustacean Biology* 2011, **31:**468-484.

13. Zierold T, Montero-Pau J, Hanfling B, Gomez A: **Sex ratio, reproductive mode and genetic diversity in *Triops cancriformis*.** *Freshwater Biology* 2009, **54:**1392-1405.

14. Bernard H: **Hermaphroditism of the Apodidae.** *Nature* 1891, **43:**343-344.

15. Longhurst AR: **A review of the Notostraca.** *Bulletin of the British Museum (Natural History)* 1955, **3:**1-57.

16. Longhurst AR: **Reproduction in Notostraca (Crustacea).** *Nature* 1954, **173:**781-782.

17. Zierold T, Hanfling B, Gomez A: **Recent evolution of alternative reproductive modes in the 'living fossil' *Triops cancriformis*.** *BMC Evolutionary Biology* 2007, **7**.

18. Mitsumoto H, Yahata K: **Evidence of cross-fertilization in a gonochoric population of the tadpole shrimp *Triops numidicus* (Crustacea : Branchiopoda : Notostraca).** *Zoological Science* 2006, **23:**1109-1113.

19. SunoUchi N, Sasaki F, Chiba S, Kawata M: **Morphological stasis and phylogenetic relationships in tadpole shrimps, *Triops* (Crustacea: Notostraca).** *Biological Journal of the Linnean Society* 1997, **61:**439-457.

20. Garcia-Velazco H, Obregon-Barboza H, Rodriguez-Jaramillo C, Maeda-Martinez AM: **Reproduction of the tadpole shrimp *Triops* (Notostraca) in Mexican waters.** *Current Science* 2009, **96:**91-97.

21. Murugan G, Maeda-Martinez AM, Obregon-Barboza H, Hernandez-Saavedra NY: **Molecular characterization of the tadpole shrimp *Triops* (Branchiopoda : Notostraca) from the Baja California Peninsula, Mexico: New insights on species diversity and phylogeny of the genus.** *Hydrobiologia* 2002, **486:**101-113.

22. Sassaman C: **Sex-ratio variation in female-biased populations of notostracans.** *Hydrobiologia* 1991, **212:**169-179.

23. Machado M, Cristo M, Reis J, Cancela L: **Biological data on *Triops cancriformis mauritanicus* (Ghigi, 1921) and *Cyzicus grubei* (Simon, 1886)—crustacea, branchiopoda—in SW Portugal temporary ponds.** *Limnetica* 1999, **16:**1-7.

24. Stephan S: **Mannchen von *Lepidurus apus* (Notostraca, Crustacea) in der Ruhstadter Elbtalaue.** *Abhandlungen und Berichte fur Naturkunde* 2008, **31:**47-57.

25. Simon E: **Étude sur les Crustacés du sous-ordre des Phyllopodes.** *Annales de la Société entomologique de France* 1886, **6:**393-460.

26. Simon E: **Étude sur les Crustacés du sous-ordre des Phyllopodes.** In.; 1886: 393-460.

27. Linder F: **Contributions to the Morphology and the Taxonomy of the Branchiopoda Notostraca, with Special Reference to the North American Species.** *Proceedings of the United States National Museum* 1952, **102:**1-69.

28. Bushnell JH, Byron ER: **Morphological variability and distribution of aquatic invertebrates (principally Crustacea) from the Cumberland Peninsula and Frobisher Bay regions, Baffin Island, NWT, Canada.** *Arctic and Alpine Research* 1979, **11:**159-177.

29. Wojtasik B, Brylka-Wolk M: **Reproduction and genetic structure of a freshwater crustacean *Lepidurus arcticus* from Spitsbergen.** *Polish Polar Research* 2010, **31:**33-44.

30. Saunders JF: **A REDESCRIPTION OF LEPIDURUS-BILOBATUS PACKARD (CRUSTACEA, NOTOSTRACA).** *Transactions of the American Microscopical Society* 1980, **99:**179-186.

31. King JL, Hanner R: **Cryptic species in a "Living Fossil" lineage: Taxonomic and phylogenetic relationships within the genus *Lepidurus* (Crustacea : Notostraca) in North America.** *Molecular Phylogenetics and Evolution* 1998, **10:**23-36.

32. Packard A: **New phyllopod crustaceans.** *American Naturalist* 1875, **9:**311-312.

33. Lynch JE: ***Lepidurus lemmoni* Holmes: a redescription with notes on variation and distribution.** *Transactions of the American Microscopical Society* 1966, **85:**181-192.

34. Kuller Z, Gasith A: **Comparison of the hatching process of the tadpole shrimps *Triops cancriformis* and *Lepidurus apus lubbocki* (Notostraca) and its relation to their distribution in rain-pools in Israel.** *Hydrobiologia* 1996, **335:**147-157.

35. Scanabissi F, Mondini C: **A survey of the reproductive biology in Italian branchiopods.** *Hydrobiologia* 2002, **486:**263-272.

36. Ahl J: **Factors affecting contributions of the tadpole shrimp, *Lepidurus packardi*, to its oversummering egg reserves.** *Hydrobiologia* 1991, **212:**137-143.

37. Margraf J, Maass B: **Zur Ökologie der temporären Süßwasserflachseen des Tafelberges „Giara di Gesturi" auf Sardinien.** *Spixiana* 1982, **5:**69-99.
